# The roles of innate and acquired immune responses on HTLV-I infection

**DOI:** 10.1186/1742-4690-8-S1-A100

**Published:** 2011-06-06

**Authors:** Mari Kannagi, Shuichi Kinpara, Atsuhiko Hasegawa, Ayako Takamori, Yukiko Shimizu, Atae Utsunomiya

**Affiliations:** 1 Department of Immunotherapeutics, Tokyo Medical and Dental University, Graduate School, Tokyo, Japan; 2 Department of Hematology, Imamura Bun-in Hospital, Kagoshima, Japan

## 

The level of HTLV-I expression is low in vivo. We found that recombinant interferon (IFN)-α and β suppressed viral expression in HTLV-I-infected cells. Non-lymphoid stromal cells also suppressed HTLV-I expression through type-I IFNs. The suppression was reversible after isolation of infected cells from the source of IFNs, mimicking the status of viral expression in freshly isolated ATL cells which is rapidly induced in culture. Nevertheless, HTLV-I-infected individuals maintain acquired immune responses against HTLV-I such as antibodies and cytotoxic T lymphocytes (CTLs), indicating the presence of HTLV-I proteins in vivo. Analysis on Tax-specific CTLs revealed that they were activated in HAM/TSP but unresponsive in ATL patients. We found that a subpopulation of HTLV-I carriers at asymptomatic stage exhibited impaired Tax-specific T-cell response and elevated HTLV-I proviral load. This combination is a feature of ATL and likely to be an underlying risk of ATL. Collectively, HTLV-I is doubly controlled by acquired and innate immunity; HTLV-I-specific CTLs eliminate infected cells, and IFNs suppress viral expression. Both would contribute the reduction of viral pathogenesis, while the efficiency of CTLs could be partial because of limited viral expression. An increase in viral expression would activate CTLs but also accelerate inflammation. When the viral expression is well-controlled, viral pathogenesis may not be apparent until infected cell clones with a malignant phenotype finally emerge, which may occur earlier without proper CTL responses. Diversity in innate and acquired immune responses among individuals might be important determinants of disease manifestation in HTLV-I infection.

